# Supplementary material for: The Evolution and Characterization of the RNA Interference Pathways in Lophotrochozoa
Source: Genome Biol Evol. 2024 May 7;16(5):evae098. doi: 10.1093/gbe/evae098 (PMC11114477; doi:10.1093/gbe/evae098)
Supplement: evae098_Supplementary_Data [file evae098_supplementary_data.zip › Supplementary_materials.zip!/selected_miRNAs/NoGe_selected_miRNAs/known_miRNA_families_pdf_files/NMRB01000470.1_Notospermus_geniculatus_strain_Ushimado_scaffold470,_whole_genome_shotgun_sequence_14117.pdf]

Provisional ID	:	NMRB01000470.1_Notospermus_geniculatus_strain_Ushimado_scaffold470,_whole_genome_shotgun_sequence_14117
Score total	:	258
Score for star read(s)	:	3.9
Score for read counts	:	249.9
Score for mfe	:	1.1
Score for randfold	:	0
Score for cons. seed	:	3
Total read count	:	502
Mature read count	:	487
Loop read count	:	0
Star read count	:	15

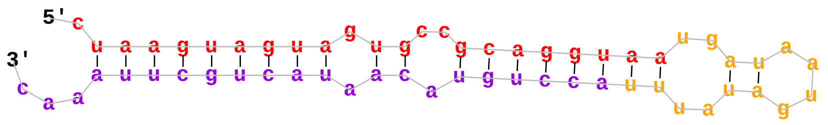

Genome coordinates: NMRB01000470.1 355690-355749

pre-miRNA sequence:
CUAAGUAGUAGUGCCGCAGGUAACUAAGUAGUAGUGCCGCAGGUAAUGAUAAUGAUAUUUACCUGUACAAUACUGCUUAAAC

Mature sequence: CUAAGUAGUAGUGCCGCAGGUAA

Aligned mature sequence of the *N. geniculatus* pre-miRNA with the top 5 hits from the BLAST search against the pre-miRNA mirGeneDB set:

```
                               *********************
Nge_Mir-252-P1e               cuaaguaguagugccgcagguaa
Cte-Mir-252-P1_5p             cuaaguaguagugccgcaggua-
Pfl-Mir-252-P1_5p             cuaaguaguagugccgcagguaa
Csc-Mir-252-P1_5p             -uaaguaguagugccgcagguaa
Sko-Mir-252-P1_5p             cuaaguaguagugccgcagguaa
Lpo-Mir-252-P1e_5p            cuaaguaguagugccgcagguaa
```
